# Supplementary material for: Quality of Life in Myasthenia Gravis and Correlation of MG-QOL15 with Other Functional Scales
Source: J Clin Med. 2022 Apr 14;11(8):2189. doi: 10.3390/jcm11082189 (PMC9025772; doi:10.3390/jcm11082189)
Supplement: Supplementary file 1 [file jcm-11-02189-s001.zip › jcm-1645532-supplementary.pdf]

Supplementary Materials

This appendix has been provided by the authors to give readers additional information about their work.

Title: "Quality of life in myasthenia gravis and correlation of MG-QOL15 with other functional sclaes".

Authors names and affiliations:

Laura Diez Porras[1], Christian Homedes[1], Maria Antonia Alberti[1,2], Valentina Velez Santamaria[1,2] and Carlos Casasnovas[1,2,3,*]

1. Neurometabolic Diseases Group. Bellvitge Biomedical Research Institute (IDIBELL). 199 Granvia de l'Hospitalet, 08908 L'Hospitalet de Llobregat, Barcelona
2. Neuromuscular Unit. Department of Neurology. Bellvitge University Hospital, Feixa Llarga street n/n, 08907 L'Hospitalet del Llobregat (Barcelona), Spain
3. Center for Biomedical Research on Rare Diseases (CIBERER), ISCIII, 3-5 Monforte de Lemos. Pabellón 121. 28029 Madrid Spain

Corresponding author: Carlos Casasnovas Pons.

E-mail: carloscasasnovas@bellvitgehospital.cat

Table S1. Inclusion and exclusion criteria

Inclusion criteria	Exclusion criteria
> 18 years	≤18 years
Electrodiagnostic criteria of MG and/or anti-RAch	Pregnant or breatfeeding women
MG class IIa to V of the MGFA clinical classification system	MG type I of the MGFA
Patients with poor response to pyridostigmine that require immunomodulatory therapy for the first time to treat the MG	Patients with hypersensitivity or contraindication to receive IVIg: serum IgA deficiency
	History of previous severe adverse event to IVIg
	Presence of diseases that could contraindicate or interfere with the treatment of oral prednisone or IVIG
	Patients who have a significative worsening in their MG after the IgIV infusion and before the prednisone initiation

Adapted from "Intravenous immunoglobulins may prevent prednisone-exacerbation in myasthenia gravis," by Díez-Porras L, Homedes C, Alberti MA, Vélez-Santamaría V, Casasnovas C, 2020, Scientific Reports, 10, 1-8. All rights reserved [2020] by Scientific Reports. Adapted with permission of the author [1].

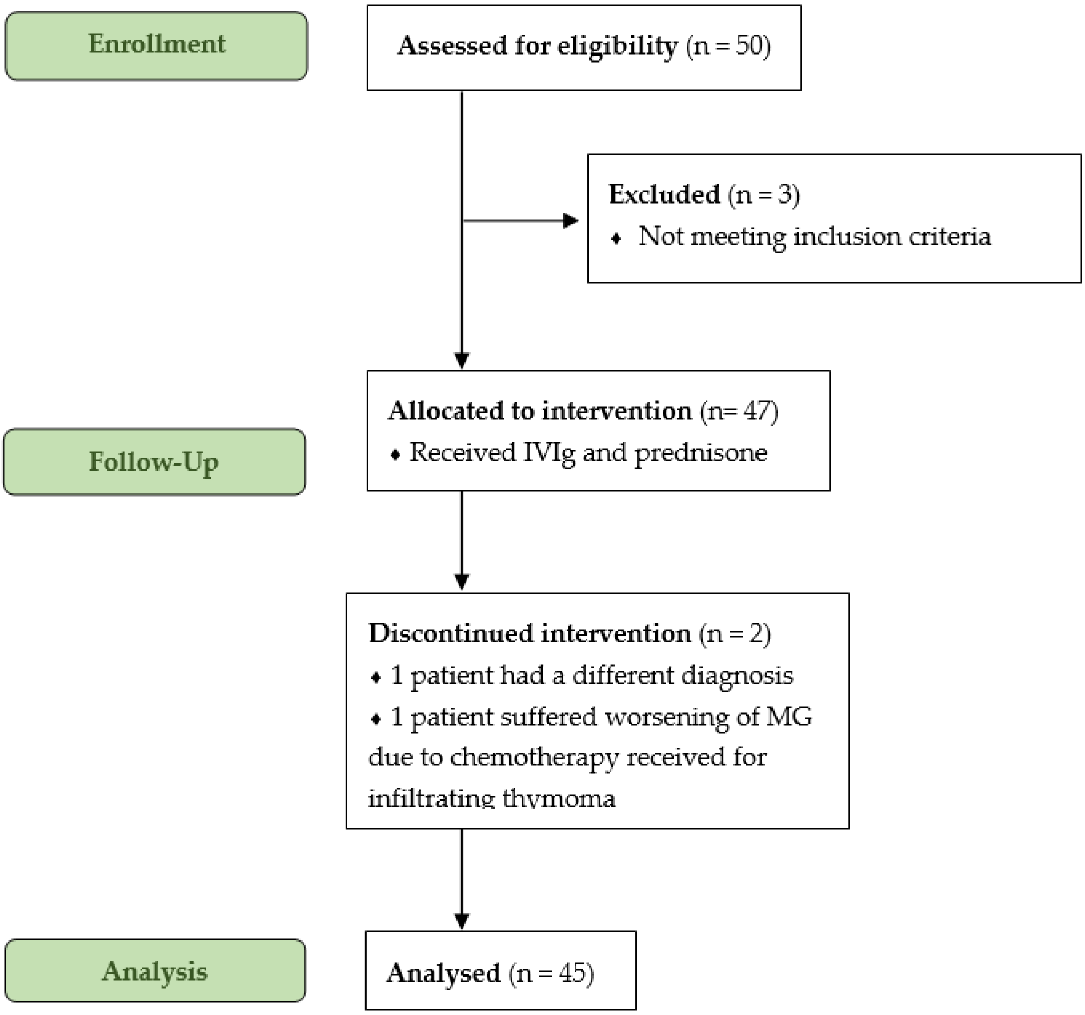

Figure S1. COSORT flow diagram.

Reference:

1. Díez-Porras L, Homedes C, Alberti MA, Vélez-Santamaría V, Casasnovas C. Intravenous immunoglobulins may prevent prednisone-exacerbation in myasthenia gravis. Sci Rep 2020;10:13497. https://doi.org/10.1038/s41598-020-70539-4.
